# Eating Habits, Physical Activity, Body Composition and Cardiorespiratory Fitness in University Students: A Cross- Sectional Study

**DOI:** 10.3390/nu17193166

**Published:** 2025-10-07

**Authors:** Edyta Kwilosz, Monika Musijowska, Katarzyna Badora-Musiał, Emilian Zadarko, Maria Zadarko-Domaradzka

**Affiliations:** 1 Institute of Health, State University of Applied Sciences in Krosno, 38-400 Krosno, Poland; 2 Faculty of Health Sciences, Institute of Public Health, Jagiellonian University Medical College, 31-008 Kraków, Poland; kasia.badora@uj.edu.pl; 3 Faculty of Physical Culture Sciences, College Medical, University of Rzeszów, 35-959 Rzeszów, Poland; ezadarko@ur.edu.pl (E.Z.); mzadarko@ur.edu.pl (M.Z.-D.)

**Keywords:** eating disorders (EDs), physical activity, cardiorespiratory fitness (CRF), bioelectrical impedance analysis (BIA), students

## Abstract

**Introduction**: Unhealthy eating habits combined with low levels of physical activity and cardiorespiratory fitness pose a serious threat to the health of young people. The aim of this research was to determine the relationship between selected components of body composition, the occurrence of eating disorders, and cardiorespiratory fitness (CRF) and physical activity levels among university students. **Material and Methods**: This study was conducted among 254 students at a university in Poland. It included the measurements of body height, body composition analysis using bioelectrical impedance analysis (BIA), and assessment of cardiorespiratory fitness (CRF). This research also employed the *My Eating Habits* (MEH) questionnaire and the short version of the *International Physical Activity Questionnaire* (IPAQ-SF). **Results**: Based on body fat percentage (BF%), nearly one-fifth (19.69%) of participants were classified as obese. According to the body mass index (BMI), over one-third had excess body weight (overweight 24.02%, obesity 10.24%), while 6.7% were underweight. Eating disorders were significantly more prevalent in women (*p* = 0.0002). A significant relationship was observed between eating disorders and BMI, muscle mass (MM%), skeletal muscle mass (SMM%), body fat (BF%), and visceral fat (VFATL). Higher BMI, BF%, and VFATL were associated with a greater risk of developing eating disorders. Emotional overeating was significantly less common among individuals with normal body weight compared to those who were underweight or overweight. No statistically significant associations were found between students’ physical activity levels and eating habits. However, cardiorespiratory fitness (CRF) was significantly negatively correlated with the presence of eating disorders. **Conclusions**: Understanding the relationship between components of body composition, eating disorders, physical activity levels, and cardiorespiratory fitness is crucial for designing effective interventions that promote a healthier lifestyle and psychological well-being among university students.

## 1. Introduction

Eating disorders (EDs) represent a major public health issue among young people worldwide. The prevalence of EDs among university students varies depending on the region and research methodology, with reported rates ranging from 12.5% to 38.4% [1,2,3,4]. This phenomenon is more common among women [4,5,6], often driven by unrealistic body image ideals. However, higher rates of obesity have been observed in individuals at risk of EDs, including both men and women [7].

The complications associated with disordered eating can be severe, which underscores the importance of early identification to prevent long-term health consequences [8]. Research shows that components of body composition (aside from fat-free mass) and cardiorespiratory fitness (CRF) mediate the relationship between eating disorder risk and bone health in young adults [9]. However, the consequences of EDs extend far beyond the skeletal system and involve other key aspects of somatic health. Recent evidence shows that unhealthy eating habits, such as irregular meal patterns, skipping breakfast, late-night eating, and frequent consumption of energy drinks, are highly prevalent among university students and young adults. These behaviors are associated with adverse outcomes, including higher BMI and fat mass, lower fat-free mass, impaired cellular health reflected by reduced phase angle, and elevated blood pressure and heart rate. Moreover, sex-specific effects have been observed, such as the link between late-night eating and higher BMI in women or the association between high salt intake and lower phase angle in men. These findings highlight the broad somatic consequences of disordered eating, extending beyond bone health and increasing metabolic and cardiovascular risks in young adults [10].

Other studies indicate an increased cardiovascular risk in young patients with mental disorders, including EDs. A large nationwide cohort study demonstrated that individuals aged 20–39 years with psychiatric disorders (such as depression, bipolar disorder, schizophrenia, anxiety disorders, or eating disorders) exhibited a higher risk of myocardial infarction (MI) and ischemic stroke compared to their peers, despite not presenting with worse baseline metabolic profiles or unhealthier lifestyle behaviors. Specifically, eating disorders were independently associated with increased risk of MI (HR 1.97; incidence rate 49.8 vs. 33.4 per 100,000 person-years) [11].

EDs may lead to both a significant decrease and an excessive increase in physical activity levels. Individuals highly focused on physical performance may develop unhealthy eating behaviors due to the pressure to maintain a specific body composition [12,13,14]. This relationship tends to be more pronounced in adolescents with higher BMI values [15].

Most studies clearly indicate that moderate, structured physical activity has a protective effect against the development of EDs. Moderate, structured physical activity also promotes mental health, contributes to increased muscle mass, improves phase angle (a marker of cellular health) and reduces body fat percentage [16,17,18].

Many students lead a sedentary lifestyle, which—combined with poor dietary habits and low levels of physical activity—poses a serious threat to their health [19,20].

The aim of the present study was to examine the relationship between selected body composition components, the occurrence of eating disorders, and CRF and physical activity levels among university students.

The relevance of this topic stems from not only by the need to obtain essential epidemiological data, but also by the practical goal of designing effective health promotion strategies in academic settings. Academic stress, social pressure, and cultural body image norms may increase the risk of developing EDs, while low physical activity levels and low CRF, as well as poor body composition at a young age, raise the risk of obesity, type 2 diabetes, hypertension and other chronic conditions later in life.

There is a lack of studies that integrate all these factors within a single population—especially among university students, who represent a unique group at increased health risk and are often overlooked in public health policies.

## 2. Materials and Methods

This research followed a cross-sectional design and was conducted at the end of the winter semester of the 2024/2025 academic year among first-year students of the State University of Applied Sciences in Krosno, Poland. The study group consisted of full-time adult students from various academic programs, including Internal Security, English Philology, Computer Science, Internet Marketing, Mechanics and Machine Construction, Nursing, Emergency Medical Services, Physical Education, and Management. The group size carefully calculated to ensure representativeness of the first-year student population.

A total of 254 students participated in this study, with females comprising the majority (51.97%). Regarding the place of residence, 65.75% of the participants lived in rural areas. The largest group was Nursing (23.62%) (Table 1).

### 2.1. Research Procedure

All measurements were performed by a trained research team in accordance with measurement procedures. Each participant completed the questionnaires independently after receiving detailed instructions from the research team, followed by somatic measurements and a cardiorespiratory fitness (CRF) test. To minimize potential sources of bias, participants were recruited from multiple faculties and academic years to enhance representativeness. Body composition and cardiorespiratory fitness (CRF) were measured using standardized protocols and calibrated equipment, with all assessments performed by the trained research team to ensure consistency, accuracy, and reliability.

### 2.2. Height and Body Composition Measurements

Body height was measured using a SECA 213 stadiometer (Seca GmbH & Co. KG, Hamburg, Germany), with an accuracy of 0.1 cm. Body mass and its components were assessed using a segmental body composition analyzer, TANITA MC-780 S MA (Tanita Corporation, Tokyo, Japan). The following components were analyzed: body fat percentage (BF%), muscle mass percentage (MM%), visceral fat level (VFATL), skeletal muscle index (SMI), phase angle (PhA), fat-free mass (FFM), and skeletal muscle mass (SMM). The measurements were obtained with participants wearing light sportswear and no footwear, in order to minimize the influence of clothing on the results.

Body mass index (BMI) was automatically calculated by the analyzer based on the entered data: height, date of birth, and gender. In accordance with WHO guidelines, BMI was classified as follows: <18.5—underweight, 18.5–24.9—normal weight, ≥25—overweight and ≥30—obesity [21,22]. For the purposes of this study, obesity was defined as BF% ≥ 35% in women and % ≥25% in men [23,24].

### 2.3. Assessment of Eating Habits

To assess eating behaviors, the My Eating Habits Questionnaire (MEH), developed by Ogińska-Bulik and Putyński [25], was used. This standardized tool was designed for older adolescents and adults to identify the presence of eating disorders primarily associated with or leading to excessive body weight. The questionnaire consisted of 30 statements, to which participants responded based on their own dietary habits Diagnostic responses were assigned a score of 1 (i.e., ‘NO’ for items 2, 5, 13, 17, and 23, and ‘YES’ for all remaining items), whereas non-diagnostic responses were assigned a score of 0. A high total score indicated disordered eating patterns, such as habitual overeating, emotional overeating, or dietary restrictions. The internal consistency of the questionnaire, measured using Cronbach’s alpha, was 0.89 [25,26].

### 2.4. Physical Activity Measurement

To assess physical activity levels, the short version of the International Physical Activity Questionnaire (IPAQ-SF) was used. This well-validated tool was developed to assess adult populations. It collected data on physical activity undertaken during the past seven days. The results allowed classification of physical activity into low, moderate, or high intensity levels, as well as sedentary behavior [27,28].

### 2.5. Assessment of Cardiorespiratory Fitness

Cardiorespiratory fitness was assessed using the multistage 20 m shuttle run test (Beep Test). The main outcome variable of the test is the distance completed (in meters), whereby longer distances are indicative of superior cardiorespiratory capacity. Participants were required to run back and forth between two lines set 20 m apart, keeping pace with auditory signals emitted from a standardized audio recording. The running speed increased progressively by 0.5 km/h at one-minute intervals. Participants continued the test until exhaustion or inability to maintain the pace according to the audio cues [26,29].

### 2.6. Inclusion Criteria

The inclusion criteria for this study were as follows: aged 18 years or older, fully completed questionnaire, good health permitting normal physical activity in the week preceding the survey and measurements. Over 300 students volunteered to participate; however, the final analysis included results from 254 students (132 women and 122 men) who completed the entire testing protocol. The selection of this group was influenced by challenges faced by students such as irregular class schedules, limited access to healthy food, and stress. Many participants were expected to exhibit improper eating habits and varying levels of physical activity. Students, especially those in sedentary study programs, form a key demographic for interventions promoting a healthier lifestyle.

### 2.7. Ethical Considerations

This study was conducted following the guidelines of the Declaration of Helsinki and was approved by the Bioethics Committee (Resolution No. 2/24 KB PANS in Krosno). Each participant gave informed consent, was assured of anonymity, and was informed that the data collected would be used solely for scientific purposes. Questionnaires were distributed personally, with clear instructions on how to answer and without time limits. Participants could withdraw from the study at any time. The chosen tools allowed analysis of the variables defined in this study.

### 2.8. Statistical Analysis

Statistical analysis included frequency analyses, calculating counts, and percentages. For quantitative variables related to the My Eating Habits Questionnaire (MEH), as well as measures of physical activity and cardiorespiratory fitness, descriptive statistics—including mean, median, minimum, maximum, standard deviation, and coefficient of variation—were calculated. The Shapiro–Wilk test was used to examine the empirical distribution of variables. Depending on the results, appropriate parametric tests (Student’s *t*-test, ANOVA) or nonparametric tests (Mann–Whitney U test, Kruskal–Wallis test) and post hoc tests with multiple comparison were applied. Spearman’s rank correlation was used to assess relationships between quantitative variables, while the chi-square test was applied for qualitative variables. Missing data were minimal in this study. For variables with incomplete responses, cases were excluded from the specific analysis. The study results were reported in accordance with the STROBE Statement [30].

## 3. Results

Based on body fat percentage (BF%) and established standards, the majority of participants (80.31%) had a normal body fat content. The remaining part of the participants (19.69%) were classified as obese. According to BMI norms, the largest group (59.06%) of students had a normal body weight relative to their height. Overweight and obesity were found in 24.02% and 10.24% of students, respectively, with underweight representing the smallest portion (6.69%). Based on BMI, 9.84% of men and 10.61% of women were classified as obese, whereas classification based on body fat percentage (BF%) indicated obesity in 20.5% of men and 18.9% of women (Table 2).

Eating disorders were significantly more frequent among women (*p* = 0.0002). The highest average scores—indicating improper eating habits—were recorded in students of English Philology ($\bar{x}$ = 11.83), Internal Security ($\bar{x}$ = 10.90) and Nursing ($\bar{x}$ = 10.69), while the lowest scores were noted among Internet Marketing ($\bar{x}$ = 10.10) (see Table 3).

The results of the survey obtained with the MEH Questionnaire and body composition components measured by BIA are presented in Table 4. Statistically significant relationships between BMI, SMM%, MM%, BF%, and VFATL and the overall eating habits, emotional overeating and dietary restrictions were identified when the calculated correlation coefficients were analyzed. The strength of the correlations was rather weak, ranging from rS = 0.16 (VFATL vs. overall) to rS = 0.32 (BF% vs. overall, emotional overeating). The only statistically significant relationship was found between emotional overeating and BMI, but the strength of the association was low.

Based on the BMI classification, the highest mean scored were observed for individuals with obesity (12.31) and overweight (10.30), while the lowest values were noticed in groups of students who were underweight (8.47) and those with a normal BMI (8.85). The overall eating habits of the students differed significantly (*p* = 0.0067), specifically between the group with obesity and the normal BMI group. The overall level on the MEH scale was also significantly higher (*p* = 0.0031) for individuals classified as obese based on body fat percentage. In this group, the mean value was 11.58, whereas among the remaining students, the mean value was 9.02 (see Table 5).

For the individual subscales of the research tool, and for obesity defined by BMI norms and body fat percentage (BF%), no statistically significant differences were found for habitual overeating. Higher mean scores for the overeating tendency scale were observed among students with obesity. These values were 3.81 for BMI classification and 3.42 for BF%, respectively. The analysis conducted for the emotional overeating showed that, within both variables considered (BMI and BF%), there were statistically significant differences between the groups (*p* < 0.05). Considering the BMI classification, the highest average value was observed for individuals with obesity (4.77), while the lowest was observed among students with a normal weight-to-height ratio. Participants classified as obese based on BF% also showed a significantly higher level (*p* = 0.0020) on the emotional overeating scale, with a mean score of 4.48. Considering the last dimension included in the MEH questionnaire and the calculated *p*-values, the levels on the dietary restraint scale differed significantly (*p* < 0.05) between groups defined by BMI and BF%. The highest average values were observed in the obesity group and amounted to 3.73 (BMI) and 3.68 (BF%), respectively. The lowest average level on the analyzed scale was observed among underweight individuals (2.18), while among those with a normal body fat percentage, it was 2.74 (see Table 6).

The post hoc test applied to the BMI grouping variable did not clearly indicate which groups differed significantly in the level of dietary restraint; the lowest *p*-value was 0.071, found for individuals classified as underweight and those with obesity.

The analysis also examined the relationship between the participants’ physical activity and aerobic endurance with their eating habits.

The majority of the surveyed students had a moderate level of physical activity. The calculated Spearman’s rank correlation values did not show any statistically significant relationship between different types of physical activity among the students and their eating habits (see Table 7).

Only two statistically significant correlations were found in the analysis of the relationship between the results of the Beep Test (20mSRT) and the scores obtained from the MEH questionnaire, namely for overall eating habits (r_s_ = −0.17) and emotional overeating (r_s_ = −0.21). Both correlations were weak in strength and negative in direction, indicating that higher physical endurance was associated with lower levels of unhealthy eating behaviors (see Table 8).

## 4. Discussion

The relationship between body composition, disordered eating behaviors, physical activity, and cardiorespiratory endurance in university students is complex and multifaceted. A higher level of physical activity is most often associated with healthier eating habits and a more positive attitude toward nutrition, whereas body dissatisfaction—shaped by BMI values and body proportions/composition—has been identified as a significant predictor of disordered eating behaviors [31,32,33].

Research into the prevalence of obesity is of particular importance, as both overweight and obesity increase the risk of chronic diseases, including type 2 diabetes, cardiovascular disease and certain types of cancer [34].

As evidenced by data published by EUROSTAT, Europe is currently experiencing an epidemic of excess body weight. Among Europeans over the age of 16, more than half (50.6%) are classified as having excess body weight (BMI ≥ 25). The countries with the highest prevalence of overweight or obesity among men are Slovakia (69.4%), Malta (69.4%), and Croatia (69.4%). In contrast, the highest proportions among women are observed in Latvia (56.7%), Lithuania (54.9%), and Finland (54.5%) [35].

Although the Body Mass Index (BMI) is commonly employed in scientific research and provides a practical estimate of excess body weight at the population level, it is subject to several well-recognized limitations. A recent study of young adults conducted by Falbova et al. (2025) found that, despite being classified as having normal body weight according to BMI (≤ 25 kg/m^2^), 31.5% of women and 19% of men met the criteria for obesity when assessed by body fat percentage (women BF% > 28%; men BF% > 20%) [36].

Obesity among university students in Europe represents a major public health concern, primarily determined by lifestyle-related factors. According to WHO BMI classification standards, over one-third of students in the studied sample were classified as having excessive body weight (24.02% overweight; 10.24% obese). When analyzing body fat percentage, about one-fifth of participants were considered obese (19.69%).

Higher obesity rates were reported in a study conducted in Peru, where among 198 university students, 14.1% were obese and 31.7% overweight (based on BMI classification) [37]. In contrast, much lower prevalence rates were observed by Ilić et al. (2024), whose study included 2452 medical students from 14 faculties across the Western Balkans region. In this population, 12% were overweight and only 2.3% were obese [38]. In another study of 603 Spanish university students aged 18 to 28 years, the prevalence of excess body weight was reported at 14.4% [39]. In Saudi Arabia, however, a study revealed that 26.4% of students were overweight and 17.1% were identified as obese [40].

Ilić et al. (2024) reported a higher prevalence of obesity among male students [38], whereas Obirikorang et al. (2024) found that excess body weight was more common among female students [41]. In the present study, no statistically significant differences in obesity prevalence were found between men and women. When analyzing body fat percentage, 20.49% of male and 18.94% of female students were categorized as obese.

Body fat analysis using the InBody 720 analyzer, performed in Galati among 258 students aged 18–23, indicated that 38% of female students were overweight or obese. When BMI was used for classification, 52.3% of participants were found to have excessive body weight [41]. Similar results were obtained by Alrubaiee et al. (2025), who reported that 53.6% of female students were overweight or obese [42].

Both body fat percentage and the risk of developing eating disorders are negatively associated with physical activity levels [15]. A study conducted among 892 nursing students in China confirmed that disordered eating behaviors adversely affect mental health, and that increased physical activity is an effective strategy for preventing psychological distress and unhealthy eating behaviors [43]. In the current study, students with a normative body weight were significantly less likely to engage in emotional overeating compared to those who were underweight or overweight. This behavior was most prevalent among individuals classified as obese, based on both BF% and BMI assessments.

A study of 1296 students aged 18–23 in China (including anthropometric measurements, physical activity assessments, and a dietary behavior questionnaire) indicated that BMI, physical activity level, muscle mass (particularly among females), and restrained eating behavior were significant predictors of body dissatisfaction. The researchers highlighted the association between students’ dissatisfaction with their appearance, low physical activity levels, and a higher risk of disordered eating in China [44]. These findings align with the results of the present study, which showed that restrictive eating was more commonly reported among individuals with obesity (based on both BF% and BMI). This may indicate that body dissatisfaction and attempts to alter dietary habits are not always well adapted to actual physiological needs.

Research conducted among athletes also shows that higher BMI or BF% scores are significantly associated with body dissatisfaction, which, in turn, constitutes a risk factor for disordered eating behaviors [45]. Heradstveit et al. (2019), in a large population-based study of 10,172 Norwegian adolescents aged 16 to 19 years (using a physical activity questionnaire and the Eating Disturbance Screening tool—EDS-5), reported that girls exhibited significantly more symptoms of eating disorders compared to boys, as well as fewer days of physical activity per week [15]. Similarly, in the present study, eating disorders were significantly more prevalent among female students. Furthermore, statistically significant differences in eating habits were observed (*p* = 0.0067) between the group with obesity and the group with normal weight status. However, the calculated correlation coefficients did not indicate a statistically significant relationship between different types of physical activity and students’ eating behaviors.

Contrasting results were reported by Huaman-Carhaus et al. (2020), who found that in both sexes, symptoms of eating disorders were negatively associated with physical activity [37]. Heradstveit et al. (2019) also concluded that eating disorder symptoms were linked to lower physical activity levels among adolescents with higher BMI scores [15], and Coman et al. (2024) identified a positive correlation between lower physical activity and increased fat mass [40]. Findings from the present study clearly demonstrated that higher total body fat and visceral fat levels were associated with an increased risk of disordered eating.

Gieniusz-Wojczyk et al. (2021), in a study on dietary habits among Polish nurses (n = 1080), found that younger nurses (<31 years) with higher BMI values were more likely to engage in habitual overeating [46]. Similar results were reported by Ogińska-Bulik and Putyński (2000), who observed that overweight women, compared to those with normal body weight, showed a greater tendency toward emotional and overeating behavior, as well as more frequent engagement in dietary restraint behaviors [25]. In the current study, BMI, SMM%, MM%, BF% and VFATL were all correlated with overall eating habits, emotional overeating, and dietary restraint. These associations were also confirmed by Grajek et al. (2022), who found that individuals with elevated BMI, low physical activity levels, and high stress were more vulnerable to developing emotional eating patterns [47].

A study conducted in Morocco by Rahim et al. (2024) highlighted the complex interaction between body weight, health, and lifestyle. The authors concluded that the high prevalence of overweight and obesity requires continuous health education interventions [48]. In research carried out by Purkiewicz et al. (2021), the occurrence of eating disorders was strongly gender-dependent [49]. Their study demonstrated that women are more susceptible to body image distortions and disordered eating behaviors. These findings, together with the results of this study, underscore the significance of the topic. The results of this study may contribute to the development of targeted strategies aimed at promoting a healthy lifestyle among young adults using the most appropriate and evidence-based approaches.

Cardiorespiratory fitness (CRF) is an indicator of the efficiency of the cardiovascular and respiratory systems in delivering oxygen during physical activity. CRF is associated with numerous health benefits, including reduced risk of cardiovascular disease, improved mental health, and better academic performance [50,51]. Evidence suggests that university students commonly exhibit CRF below normative standards, which could negatively impact their overall health [52,53]. A higher BMI is associated with lower CRF levels, and students with overweight or obesity tend to have reduced CRF compared to their peers with normal body weight [54].

In the present study, a relationship was identified between the results of the Beep Test (20mSRT) and scores obtained from the MEH questionnaire. Statistically significant correlation coefficients were calculated for overall eating habits (r_s_ = –0.17) and emotional overeating (r_s_ = –0.21), although the strength of these associations was weak.

## 5. Conclusions

Eating disorders are influenced by multiple factors. The findings of the present study indicate that students exhibit insufficient levels of physical activity and a wide range of cardiorespiratory endurance levels. When combined with unhealthy eating habits and the high prevalence of disordered eating behaviors, these findings highlight the urgent need for comprehensive health education and the promotion of a healthy lifestyle within the academic environment. Such initiatives should be carefully tailored to the specific needs and challenges of this population.

## 6. Limitations

This study was cross-sectional in nature, and the data were collected from students from a single university based on convenience sampling, as not all invited first-year students agreed to participate, which may limit the generalizability of the results. Despite the limitations of univariate analyses, the findings provide useful insights and suggest directions for future longitudinal research. This research employed both self-report tools—which may be subject to social desirability bias, in which participants present themselves more favorably than they actually are—and objective measures, such as body composition analysis (BIA) and cardiorespiratory fitness assessment (CRF), which provided reliable and valid results.

Future research should aim to assess participants’ dietary intake more precisely, for example, through food frequency questionnaires (FFQs), and should also consider evaluating levels of body satisfaction and psychological stress.

## Figures and Tables

**Table 1:** Full descriptive statistics of the study group.

Parameters	Descriptive Statistics	N (%)
Gender	Female	132 (51.97)
	Male	122 (48.03)
Place of residence	Urban	87 (34.25)
	Rural	167 (65.75)
Field of study	Internal Security	20 (7.87)
	English Philology	40 (15.75)
	Computer Science	19 (7.48)
	Internet Marketing	33 (12.99)
	Mechanics & Machine Construction	15 (5.91)
	Nursing	60 (23.62)
	Emergency Medical Services	13 (5.12)
	Physical Education	30 (11.81)
	Management	24 (9.45)

N—group size.

**Table 2:** Frequency of normative and non-normative body weight in the studied student group based on BMI and body fat percentage, by gender.

Variable	Variant	Women N (%)	Men N (%)	Total N (%)
BMI [kg/m^2^]	Underweight	15 (11.36)	2 (1.64)	17 (6.69)
	Normal	77 (58.33)	73 (59.84)	150 (59.06)
	Overweight	26 (19.7)	35 (28.69)	61 (24.02)
	Obesity	14 (10.61)	12 (9.84)	26 (10.24)
Body Fat [%]	No Obesity	107 (81.06)	97 (79.51)	204 (80.31)
	Obesity	25 (18.94)	25 (20.49)	50 (19.69)

N—group size.

**Table 3:** Numerical characteristics of the overall My Eating Habits Questionnaire (MEH) scale by selected qualitative variables.

Variable	Variant	N	$\bar{x}$	SD	Min	Median	Max	R	CV	*p*-Value
Gender	Women	132	10.69	5.35	1.00	10.00	27.00	26.00	50.07	0.0002 *
	Men	122	8.27	4.42	1.00	7.00	22.00	21.00	53.49	
Field of Study	Internal Security	20	10.90	5.98	5.00	10.00	26.00	21.00	54.88	
	English Philology	40	11.83	5.24	3.00	12.00	25.00	22.00	44.29	0.0306 *
	Computer Science	19	8.84	4.91	1.00	7.00	21.00	20.00	55.57	
	Internet Marketing	33	7.91	3.91	1.00	7.00	17.00	16.00	49.41	
	Mechanics & Machine Construction	15	8.20	5.10	1.00	8.00	22.00	21.00	62.22	
	Nursing	60	10.10	5.75	1.00	9.00	27.00	26.00	56.94	
	Emergency Medical Services	13	7.92	4.84	2.00	7.00	17.00	15.00	61.07	
	Physical Education	30	8.97	4.25	2.00	9.00	17.00	15.00	47.36	
	Management	24	8.29	3.16	1.00	8.00	15.00	14.00	38.05	
Total		254	9.53	5.07	1.00	9.00	27.00	26.00	53.16	

N—group size, $\bar{x}$—average value, SD—standard deviation, Min—minimum, Me—median, Max—maximum, R—range, CV—coefficient of variation, *—statistically significant (α = 0.05).

**Table 4:** Correlation of Selected Body Composition Components and My Eating Habits (MEH) Among Students.

	Overall	Habitual	Emotional	Restrictions
BMI	0.27 *	0.13 *	0.26 *	0.23 *
SMI	−0.02	0.03	−0.04	−0.02
SMM %	−0.20 *	−0.07	−0.24 *	−0.16 *
MM %	−0.21 *	−0.08	−0.24 *	−0.18 *
BF %	0.32 *	0.11	0.32 *	0.29 *
FFM	0.01	0.08	−0.03	−0.03
VFATL	0.16 *	0.06	0.18 *	0.15 *
P_ANGLE	−0.06	0.06	−0.12	−0.09

BMI—Body Mass Index, SMI—Skeletal Muscle Mass Index, SMM—Skeletal Muscle Mass, MM %—Muscle Mass Percentage, BF%—Body Fat Percentage, FFM—Fat-Free Mass, VFATL—Visceral Fat Level, M_AGE—Biological Age, P_ANGLE—Phase Angle, *—Statistical Significance (α = 0.05).

**Table 5:** Descriptive statistics for the overall MEH scale in relation to BMI classification and obesity defined by body fat percentage (BF%).

Variable	Variant	N	$\bar{x}$	SD	Min	Median	Max	R	CV	*p*-Value
BMI	Underweight	17	8.47	4.69	3.00	8.00	20.00	17.00	55.39	0.0067 *
	Normal	150	8.85	4.81	1.00	8.00	27.00	26.00	54.29	
	Overweight	61	10.30	5.29	1.00	9.00	25.00	24.00	51.41	
	Obesity	26	12.31	526	4.00	11.50	23.00	19.00	42.73	
BF%	No Obesity	204	9.02	4.75	1.00	8.00	26.00	25.00	52.62	0.0031 *
	Obesity	50	11.58	5.81	1.00	11.00	27.00	26.00	50.14	
	Total	254	9.53	5.07	1.00	9.00	27.00	26.00	53.16	

N—group size, $\bar{x}$—average value, SD—standard deviation, Min—minimum, Me—median, Max—maximum, R—range, CV—coefficient of variation, *—statistically significant (α = 0.05).

**Table 6:** Descriptive statistics for the MEH scale in relation to BMI classification and obesity determined by body fat percentage (BF%).

Variable	Variant	N	Habitual Overeating				Emotional Overeating				Dietary Restraint			
			$\bar{x}$	SD	CV	*p*-Value	$\bar{x}$	SD	CV	*p*-Value	$\bar{x}$	SD	CV	*p*-Value
BMI	Underweight	17	2.88	2.23	77.46	0.282	3.41	2.32	68.00	0.003 *	2.18	1.91	87.83	0.036 *
	Normal	150	2.95	2.23	75.49		3.13	2.09	66.73		2.77	2.10	75.56	
	Overweight	61	3.28	2.18	66.62		3.87	2.25	58.27		3.15	2.26	71.94	
	Obesity	26	3.81	2.50	65.61		4.77	2.35	49.37		3.73	2.07	55.48	
BF %	No obesity	204	3.04	2.18	71.69	0.435	3.25	2.07	63.71	0.002 *	2.74	2.10	76.79	0.002 *
	Obesity	50	3.42	2.52	73.80		4.48	2.55	56.91		3.68	2.16	58.73	
	Total	254	3.11	10.00	72.28		3.49	10.00	63.64		2.92	9.00	73.31	

N—group size, $\bar{x}$—average value, SD—standard deviation, CV—coefficient of variation, *—statistically significant (α = 0.05).

**Table 7:** Descriptive statistics of eating habits based on the physical activity classification of the surveyed students.

Eating Habits	Variant	N	$\bar{x}$	SD	Min	Median	Max	R	CV	*p*-Value
Overall	Insufficient	58	8.83	1.00	7.50	27.00	26.00	4.97	56.27	0.3887
	Moderate	158	9.72	1.00	9.00	26.00	25.00	5.19	53.40	
	High	38	9.82	2.00	9.00	23.00	21.00	4.71	48.01	
	Overall	254	9.53	5.07	1.00	9.00	27.00	26.00	53.16	
Habitual	Insufficient	58	2.84	0.00	3.00	10.00	10.00	2.17	76.16	0.4556
	Moderate	158	3.13	0.00	3.00	9.00	9.00	2.25	71.81	
	High	38	3.47	0.00	3.00	8.00	8.00	2.40	69.15	
	Overall	254	3.11	10.00	0.00	2.25	3.00	10.00	72.28	
Emotional	Insufficient	58	3.09	0.00	3.00	9.00	9.00	2.09	67.66	0.3139
	Moderate	158	3.66	0.00	3.00	10.00	10.00	2.30	62.64	
	High	38	3.39	0.00	3.00	8.00	8.00	2.07	61.08	
	Overall	254	3.49	10.00	0.00	2.22	3.00	10.00	63.64	
Restraint	Insufficient	58	2.90	0.00	2.00	8.00	8.00	2.01	69.56	0.9630
	Moderate	158	2.92	0.00	2.00	9.00	9.00	2.24	76.48	
	High	38	2.95	0.00	2.00	8.00	8.00	1.97	66.91	
	Overall	254	2.92	9.00	0.00	2.14	2.00	9.00	73.31	

N—group size, $\bar{x}$—average value, SD—standard deviation, Min—minimum, Me—median, Max—maximum, R—range, CV—coefficient of variation.

**Table 8:** Correlation between the physical activity of students and their eating habits as measured by the MEH questionnaire.

Variable	Overall	Habitual	Emotional	Restraint
Distance 20mSRT	−0.17 *	−0.06	−0.21 *	−0.12
Vigorous Activity	−0.04	0.02	−0.07	−0.04
Moderate Activity	0.06	0.01	0.02	0.07
Low Activity	0.05	0.06	0.04	−0.04
Weekly Activity	0.04	0.05	−0.01	0.00
Sitting	0.08	0.02	0.10	0.09

*—statistically significant (α = 0.05).

## Data Availability

The original contributions presented in this study are included in the article. Further inquiries can be directed to the corresponding authors.
